# Dominant change pattern of extreme precipitation and its potential causes in Shandong Province, China

**DOI:** 10.1038/s41598-022-04905-9

**Published:** 2022-01-17

**Authors:** Jun Xia, Xu-yang Yang, Jian Liu, Mingsen Wang, Jiake Li

**Affiliations:** 1 School of Water Conservancy and Hydropower Engineering, Xi’an University of Technology, Xi’an, China; 2 State Key Laboratory of Water Resources and Hydropower Engineering Science, School of Water Resources and Hydropower Engineering, Wuhan University, Wuhan, China; 3 Post-Doctoral Research Center, Shandong Provincial Academician Workstation, Water Resources Research Institute of Shandong Province, Jinan, China

**Keywords:** Climate sciences, Hydrology

## Abstract

Due to global warming, global and regional extreme precipitation events occur frequently, causing severe drought and flood disasters. This has a significant impact on productivity and human life. Therefore, it is of great significance to study the characteristics of extreme precipitation and its spatiotemporal variation. In this study, we investigate the dominant variation patterns of extreme precipitation (EP), which is characterized by indices, and also analyze its potential causes in the Shandong province of China during 1961–2015 using the daily precipitation data from 123 metrological stations. The results show that there has been a dry trend in the Shandong Province in the past 55 years, that is, with the decrease in precipitation, most of the extreme precipitation index has basically showed a downward trend to varying degrees. In particular, the increase in the number of consecutive dry days (CDD) and the decrease in the number of consecutive wet days (CWD) can better explain the drought in this region. After the 1980s, the extreme precipitation index basically showed an upward trend to varying degrees, indicating that extreme precipitation events have shown an increasing trend in recent years. The spatial distribution of each extreme precipitation index generally increased from north to south. The mutation of each extreme precipitation index occurred in the 1970s and 1990s, and there was a main period of 0.9–2.2 years. In terms of influencing factors, the NINO3 area can be used as the critical sea area for the response of extreme precipitation to SSTAs in the Shandong Province. The research results are helpful to understand the temporal and spatial variations of extreme precipitation and have very important reference value for the prediction of and response to climate change and extreme events in the future.

## Introduction

In the past 100 years, the world has experienced a climate change process that is characterized by warming^1^. Climate change is more severe than originally thought. As a result of human activities, the occurrence of such extreme cases has increased and will continue to increase in the future, even when circumstances are artificially controlled as much as possible^2,3^. Therefore, in the future, extreme weather and climate events caused by global climate change will be one of the most significant challenges facing mankind^4–6^. Small changes in the climate have had a profound impact on people's lives, and these extreme climate events often have a huge adverse impact on socio-economic systems and terrestrial ecosystems^7–9^. In 2015, disasters caused financial losses of close to $100 billion worldwide and caused 23,000 fatalities^10^. The amount of precipitation has increased with the global warming. Precipitation has increased in most of the tropics, decreased in most of the subtropics, and tended to increase at high latitudes, and more frequent droughts and floods may occur in different places^11–13^. Overall, the changes of extreme precipitation are different in different regions of the world, while the frequency of heavy precipitation in regions that account for more than half of the global land area shows an increasing trend^14^. A variation trend of the total precipitation in China is not evident, and the number rainy days has decreased significantly. From the perspective of regional change, the precipitation in the Yangtze River basin has increased, the precipitation in North China has decreased, and the number of rainy days has decreased significantly. This indicates that the precipitation process will tend to be concentrated, and droughts and floods will tend to increase^15^. In the areas where the precipitation is increasing, the number of heavy precipitation events is very likely to increase^16,17^. Even if the average precipitation decreases or remains unchanged, there is still an increase in the heavy precipitation and precipitation frequency^18^. Many scholars have reported similar findings on the long-term variation characteristics of precipitation or heavy precipitation in many regions of the world^18^. The variation pattern of extreme events can be spatially differentiated^19^. Studying the characteristics of extreme precipitation in different regions is helpful for understanding the temporal and spatial variations of extreme precipitation. Understanding these characteristics has a very important reference value and significance for predicting and dealing with climate change and extreme events in the future.

In this study, the Shandong Province, which is located in the eastern coast of China, was selected as the study area to investigate the spatiotemporal variation and potential causes of extreme precipitation. The observed daily precipitation data at 123 meteorological stations from 1961 to 2015 were used. Shandong is an important agricultural production area in China and the topography is particularly complex, extreme precipitation events occur frequently due to global warming, which may lead to a shortage of available water resources, soil droughts, an increase of the harm caused by diseases and insect pests, and a reduction of agriculture and fishery production and income^20^. To the best of our knowledge, previous studies in this area were mainly focused on trends of extreme precipitation, and the density of meteorological station networks is low. Therefore, it is necessary to analyze the spatial and temporal variations of extreme precipitation and explore the influencing factors. The main objectives of this study were as follows: (1) to analyze the temporal trends and spatial distribution of the extreme precipitation indices for the Shandong Province, and (2) to discuss the response relationship between precipitation index in the Shandong Province.

## Materials and methods

### Study area

**Figure 1:**
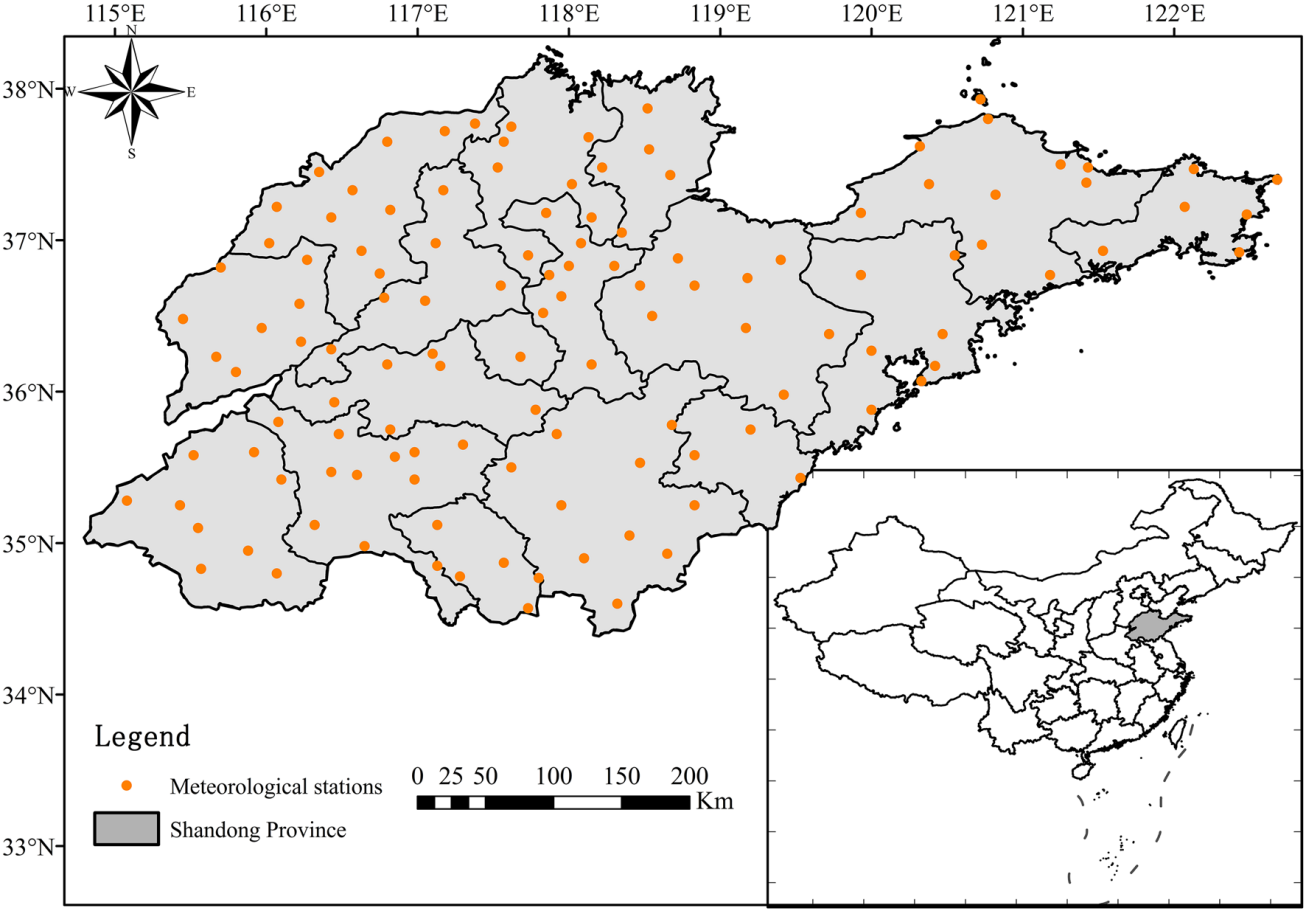
Location of meteorological stations and Shandong Province (The software used to create the maps in Figs. 1, 3 and 4 is ArcGIS10.4, URL: http://gisserver.domain.com:6080/arcgis).

The Shandong Province is one of the economically developed provinces along the eastern coast of China, which is not only a major agricultural province but also a strong industrial province. The territory consists of two parts: a peninsula and an inland, and the geographical location of the peninsula protruding into the Bohai Sea makes it rich in water vapor. Shandong Province is located between the eastern coast of China and the lower reaches of the Yellow River with latitude ranges from 34°23′ to 38°24′ N and longitude ranges from 114°47′ to 122°42′ E. With a total population of 100.7 million, this province now has jurisdiction over 16 cities and 137 counties (cities and districts) (Fig. 1). It is an important agricultural area in China, known as “the storehouse of grain, cotton and oil, and the hometown of fruit and aquatic products.” Its grain output ranks second in the country and has the largest vegetable base in the country^21^. The whole province is a typical continental monsoon climate area, with annual precipitation mainly concentrated in the summer, summer precipitation (From June to August) accounts for 62% of annual precipitation (1961–2015 average precipitation of 676.3 mm). The province is prone to flooding and waterlogging, which brings significant economic losses to agricultural and industrial production. Shandong Province is located across the Yellow River, Huaihe River, and Haihe River basins^22^. Due to the special geographical location and climate conditions, the region response to climate change is sensitive and strong. With the frequent occurrence of drought disaster, “nine out of ten years will be droughts, droughts in spring every year, and droughts and floods turn sharply” is the true portrayal of the provincial and water conditions in the Shandong Province^23^. In the dry months, there is an increase in the water deficit for agriculture, and irrigation will require larger quantities of water^24^. This has a great impact on agricultural production, which is highly dependent on irrigation. The characteristics and changes of extreme precipitation have an important impact on the agricultural production and economic development in the Shandong Province.

### Data

The precipitation data used come from the climate reference room of the National Meteorological Information Center of the China Meteorological Administration. With repeated monitoring and control, the data set is of high quality. To use the longest continuous segment of data, the daily precipitation data of 123 meteorological stations in the Shandong Province from 1961 to 2015 were selected, in which the data of 1–2 days were interpolated as the average of adjacent days, and the data of more than 3 days were interpolated by the multi-year average of the data of the same period. The daily precipitation data of 123 meteorological stations after processing were verified for homogeneity using three statistical methods: the von Neumann ratio (N), cumulative deviations (Q/n-0.5, R/n-0.5), Bayesian procedures (U, A), all of which passed the 95% confidence test.

### Methodology

#### Methods

##### (1) Trend analysis

In this study, the linear regression equation^25^ and the 5-point moving average method^25^ were used to analyze the trend characteristics of the time series. The ordinary Kriging interpolation method is used to analyze the spatial distribution of the extreme precipitation index over many years.

##### (2) Mutation analysis

In this study, three test methods were used to study the variations of the time series, namely, Mann–Kendall (M − K) tests^26,27^, cumulative anomaly tests^25^, and Pettitt tests^25^. The application problems in mutation theory research are the most active and controversial. Sometimes the detection methods used are improper, which may lead to wrong conclusions. Therefore, when determining the mutation phenomenon of a meteorological system or process, it is best to use a variety of methods for comparison and judge in combination with the actual climate performance^25^.

##### (3) Wavelet analysis method

In this paper, wavelet analysis^25^ is used to analyze the periodicity of climatic elements. Wavelet analysis is based on wavelet transform. For one-dimensional time series function, wavelet transform can be defined in Eq. (1):

1 $$W\left(\tau ,s\right)=\frac{1}{\sqrt{s}}{\int }_{-\infty }^{+\infty }f\left(t\right){\psi }^{*}\left(\left(t-\tau \right)/s\right)dt$$

where, $f\left(t\right)$ is analysis wavelet function, $\tau$ is a local time index, $s$ is a wavelet parameter, $\psi$ is a wavelet basis function, $*$ represents the conjugation of complex numbers.

In this study, Morlet wavelet is selected as the basis function of wavelet transform, and it can be defined in Eq. (2):

2 $$\psi \left(\eta \right)={\pi }^{-\frac{1}{4}}{e}^{i\omega \eta }{e}^{-\frac{1}{2}{\eta }^{2}}$$

where, $\eta$ and $\omega$ represent dimensionless time parameters and frequencies, respectively,$\eta =t/s$.

For two time series $X\left(t\right)$ and $Y\left(t\right)$ and their respective wavelet transformations ${W}^{X}\left(t,s\right)$ and ${W}^{Y}\left(t,s\right)$, the cross wavelet spectrum is defined in Eq. (3).

3 $${W}^{XY}\left(t,s\right)={W}^{X}\left(t,s\right){W}^{Y}\left(t,s\right)$$

Wavelet coherence coefficient can be calculated according to the Eq. (4).

4 $${R}^{2}\left(t,s\right)={|\langle {s}^{-1}{W}^{XY}\left(t,s\right)\rangle |}^{2}/\langle {s}^{-1}{W}^{X}\left(t,s\right)\rangle \langle {s}^{-1}{W}^{Y}\left(t,s\right)\rangle $$

where,${R}^{2}\left(t,s\right)$ is wavelet coherence coefficient; “$\langle \rangle$” represents the smoothing function of the wavelet energy spectrum smoothing at both the coordinate scale and the time scale; ${s}^{-1}$ is the conversion factor between the wavelet coherence spectrum and the energy density.

For a global wavelet spectrum (Global Wavelet Power, GWP) of a specific scale $s$, which is the time average of all local wavelet energy spectra (Wavelet Power Spectra, WPS), and it can be calculated according to the Eq. (5).

5 $${\overline{W} }^{2}\left(s\right)=\frac{1}{N}{\sum }_{n=0}^{N-1}{|{W}_{n}\left(s\right)|}^{2}$$

where, $N$ is the sample of time series.

Wavelet variance is the sum of squares of the modulus deviation of wavelet coefficients, which reflects the distribution of wave energy with scales, and can be used to determine the relative intensity of disturbances of various scales in a time series. The scale at the corresponding peak is the main time scale of the series, that is, the main period. If the time series period passes the significance level, it is a significant period. Wavelet variance can be calculated according to the Eq. (6).

6 $${S}^{2}=\frac{1}{n-1}{\sum }_{i=1}^{n}{\left({c}_{i}-\overline{u }\right)}^{2}$$

where, ${S}^{2}$ is the wavelet variance; ${c}_{i}$ is the wavelet coefficient; $\overline{u }$ is the mean value of the wavelet coefficient; $n$ is the number of wavelet coefficients. The period that passes the 95% significant test is a significant test is a significant period, and the period that fails the 95% significant test is a non-significant period.

#### Extreme precipitation indices

Both the third and fourth assessment reports of IPCC have clearly defined extreme climate events^28,29^. Extreme climate events are events with a very low probability of occurrence. For a particular location and time, the probability of occurrence usually accounts for only 10% or less of such a climate phenomenon. This definition takes into account the difference of climate in different regions, thereby avoiding the problem that the absolute intensity of events varies greatly from region to region, making it difficult to compare with a unified standard.

The World Meteorological Organization for Commission for Climatology (WMO-CCI) and Expert Team for Climate Change Detection, Monitoring, and Indices (ETCCDMI) have developed and recommended 27 climate extreme indices for comprehensive assessment of climate extremes change across a wide variety of climates. The 27 core indices include 16 temperature indices and 11 precipitation indices. In this study, we selected 11 precipitation indices for precipitation analysis (Table 1), and the precipitation indices of each station were calculated using the RclimDex software^30^. To study extreme climate events, the percentile threshold method is usually employed. When the value of the climate element is greater than the selected threshold, the climate element value can be called the extreme value, and the event is called an extreme event. Extreme precipitation events are one of the climate extreme events. In this study, the widely used percentile threshold method is used to define the extreme precipitation threshold of each station. Using the precipitation observation data from 1961 to 2000, the value corresponding to the 5th percentile is taken as the extreme minimum threshold value, and the value corresponding to the 95th percentile is taken as the extreme maximum threshold value.

**Table 1:** List of climate extreme indices for precipitation used in this study.

Classification	ID	Index name	Definition	Units
Indices related to the amount of precipitation	Rx1day	Max 1-day precipitation amount	Monthly maximum 1-day precipitation	mm
	Rx5day	Max 5-day precipitation amount	Monthly maximum consecutive 5-day precipitation	mm
	R95p	Very wet days	Annual total PRCP when RR > 95th percentile	mm
	R99p	Extremely wet days	Annual total PRCP when RR > 99^th^ percentile	mm
	SDII	Simple daily intensity index	Annual total precipitation divided by the number of wet days (defined as PRCP ≥ 1.0 mm) in the year	mm/day
	PRCPTOT	Annual total wet-day precipitation	Annual total PRCP in wet days (RR ≥ 1 mm)	mm
Indices related to the number of precipitation days	CDD	Consecutive dry days	Maximum number of consecutive days with RR < 1 mm	Days
	CWD	Consecutive wet days	Maximum number of consecutive days with RR ≥ 1 mm	Days
	R10	Number of heavy precipitation days	Annual count of days when PRCP ≥ 10 mm	Days
	R20	Number of very heavy precipitation days	Annual count of days when PRCP ≥ 20 mm	Days
	Rnn	Number of days above nn_mm	Annual count of days when PRCP ≥ nn_mm, nn is user defined threshold	Days

### Ethics declarations

This article does not contain any studies with human participants or animals performed by any of the authors.

## Results and discussion

### Temporal variation of extreme precipitation

#### Indices related to the number of precipitation days

**Figure 2:**
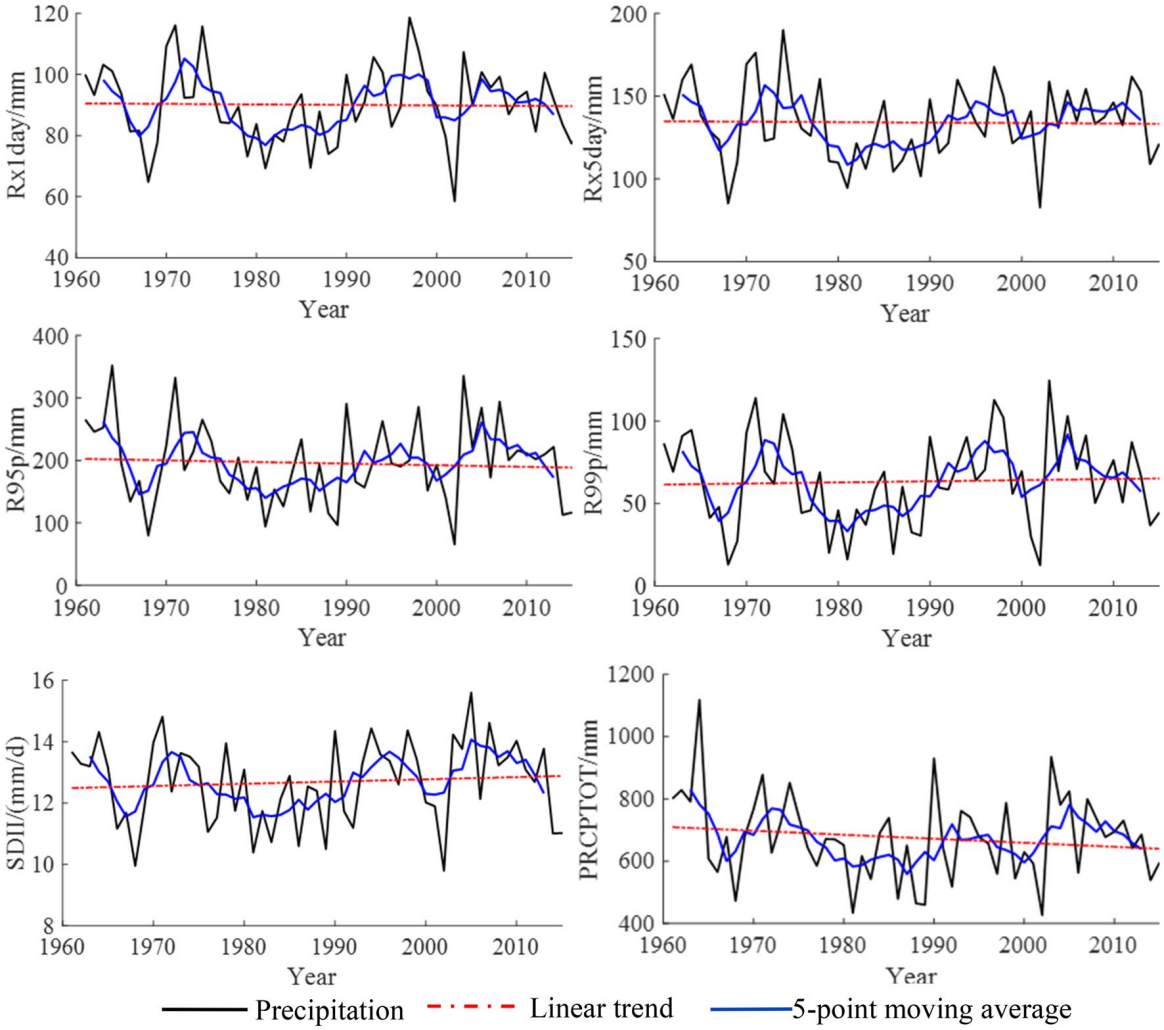
Temporal variations of indices related to the number of precipitation days in Shandong from 1961 to 2015.

The time variation trends of the extreme indices related to the number of precipitation days in the Shandong Province from 1961 to 2015 are shown in Table 2 and Fig. 2. Of the core indices related to the number of precipitation days, only the variation trend of CWD passed the significance test at the 95% confidence level. CDD had an upward trend, and its climate tendency rate was 0.62 d/10a. From the perspective of a 5-point moving average, there was a small oscillatory upward trend aside from a large fluctuation at the end of 90 s. CWD, R10, R20, and Rnn all had downward trends, and their climate tendency rates were 0.10 d/10a, − 0.29 d/10a, − 0.19 d/10a, and − 0.09 d/10a, respectively. From the perspective of a 5-point moving average, the CWD showed an oscillatory downward trend, but it has shown an upward trend since the 1980s. R10, R20, and Rnn fluctuated greatly in the 1960s and early twenty-first century.

**Table 2:** Variations of extremes precipitation indices in Shandong from 1961 to 2015.

Indices	Indices related to the amount of precipitation						Indices related to the number of precipitation days				
	Rx1day	Rx5day	R95p	R99p	SDII	PRCPTOT	CDD	CWD	R10	R20	Rnn
	mm/10a				mm/d/10a	mm/10a	d/10a				
Trend1^a^	− 0.16	− 0.28	− 2.60	0.69	0.07	− 12.88	0.62	− 0.10	− 0.29	− 0.19	− 0.09
Trend2^b^	− 0.92	− 1.65	− 2.27	− 0.70	0.11	− 14.98	1.23	− 0.13	− 0.47	− 0.20	− 0.19

^a^Trend1 represents the time variation trend of the extreme precipitation indices in this study.

^b^Trend2 represents the time variation trend of the extreme precipitation indices in the coastal areas of Huanghuai, China (including Shandong Province) (Wang 2017).

In summary, from 1961 to 2015, although the overall trend of the extreme precipitation indices has risen and fallen. The increasing trend of the extreme precipitation index and the number of rainfall days of different levels in the later periods indicate that the numbers of extreme precipitation events and extreme rainfall days in the Shandong Province are increasing. However, the increase in CDD and the decrease in CWD also indicated that the drought in the study area was aggravating. As shown in Table 2, the time variation trend of the extreme precipitation indices was consistent with the existing research results^31^.

### Spatial distribution of extreme precipitation

#### Indices related to the amount of precipitation

**Figure 3:**
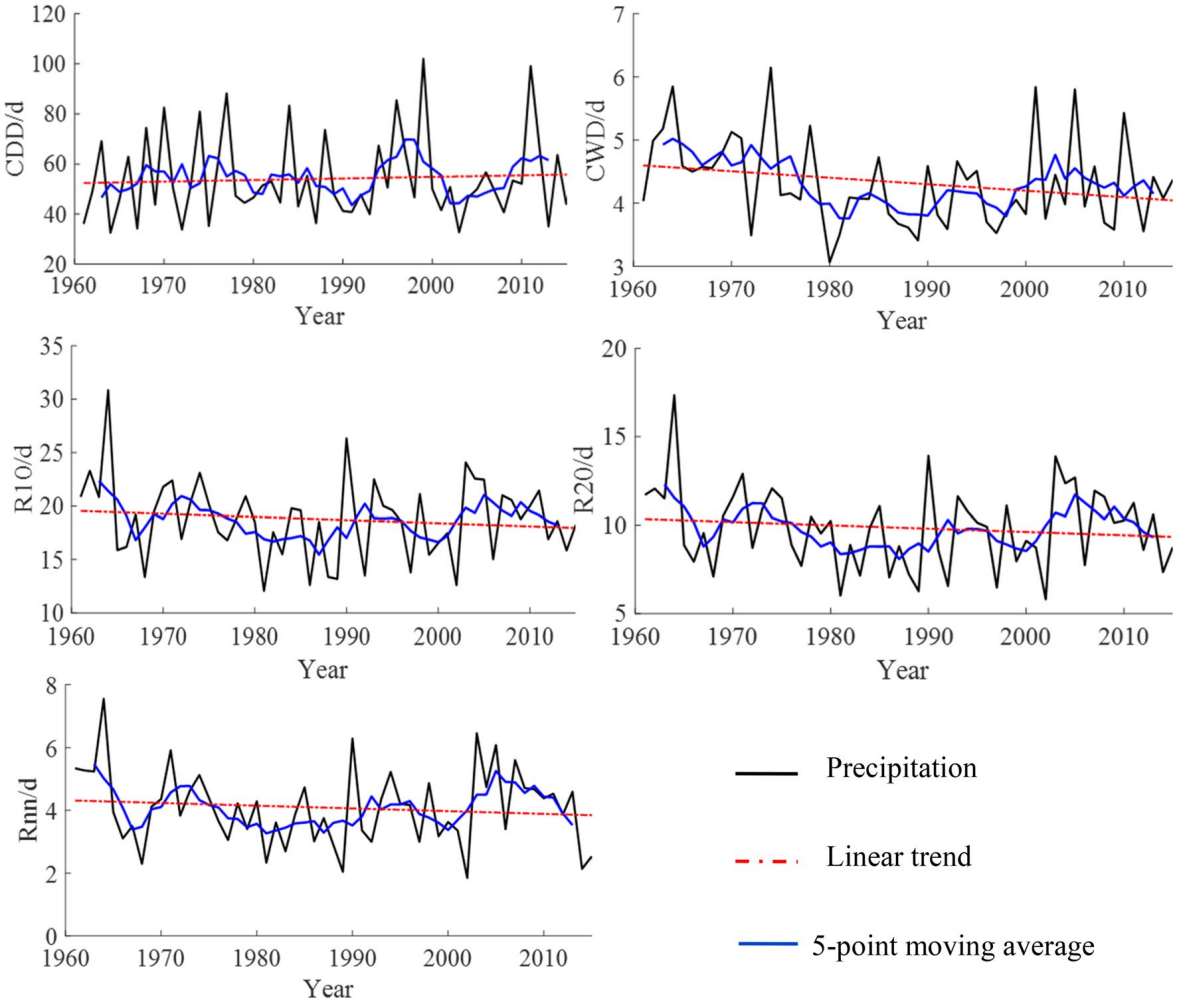
Temporal distribution of indices related to the amount of precipitation in Shandong from 1961 to 2015.

From 1961 to 2015, indices related to the amount of precipitation in the Shandong Province showed differences in their spatial distributions. Kriging interpolation was applied to the core indices of 123 stations in the Shandong Province, and the continuous spatial distribution in the region was obtained, as shown in Fig. 3. From northern to central Shandong Province, the value of Rx1day decreases from 86 to 70 mm. From central to southwestern Shandong Province, the value of Rx1day increases from 70 to 115 mm. Approximately 50% of the meteorological stations showed an upward trend, and about 95% of the stations with an upward trend passed the significance test. Approximately 93% of the stations with a downward trend passed the significance test. From the northwest to the southeast of Shandong Province, the value of Rx5day increases from 114 to 166 mm, the value of R95p increases from 157 to 252 mm. About 42% of the meteorological stations showed upward trends of Rx5day, 58% and 77% of the stations with upward and downward trends passed the significance test, respectively. About 38% of the meteorological stations for R95p showed an upward trend and failed the significance test. From the northwest to the southeast of Shandong Province, the value of R99p increases from 55 to 74 mm, and there were three high value areas in the southwest, east-central, and northeast of the Shandong Province. About 47% of the stations showed upward trends. The variation trend of this index in all of the stations failed the significance test. From the northwest to the south of Shandong Province, the value of SDII increases from 11 mm/day to 15 mm/day. About 70% of the stations showed upward trends, and the variation trend of this index at all of the sites passed the significance test. From the northwest to the south of Shandong Province, the value of PRCPTOT increases from 534 mm/day to 863 mm. Only about 11% of the stations showed an upward trend, and the variation trend of this index of all sites failed the significance test.

#### Indices related to the number of precipitation days

**Figure 4:**
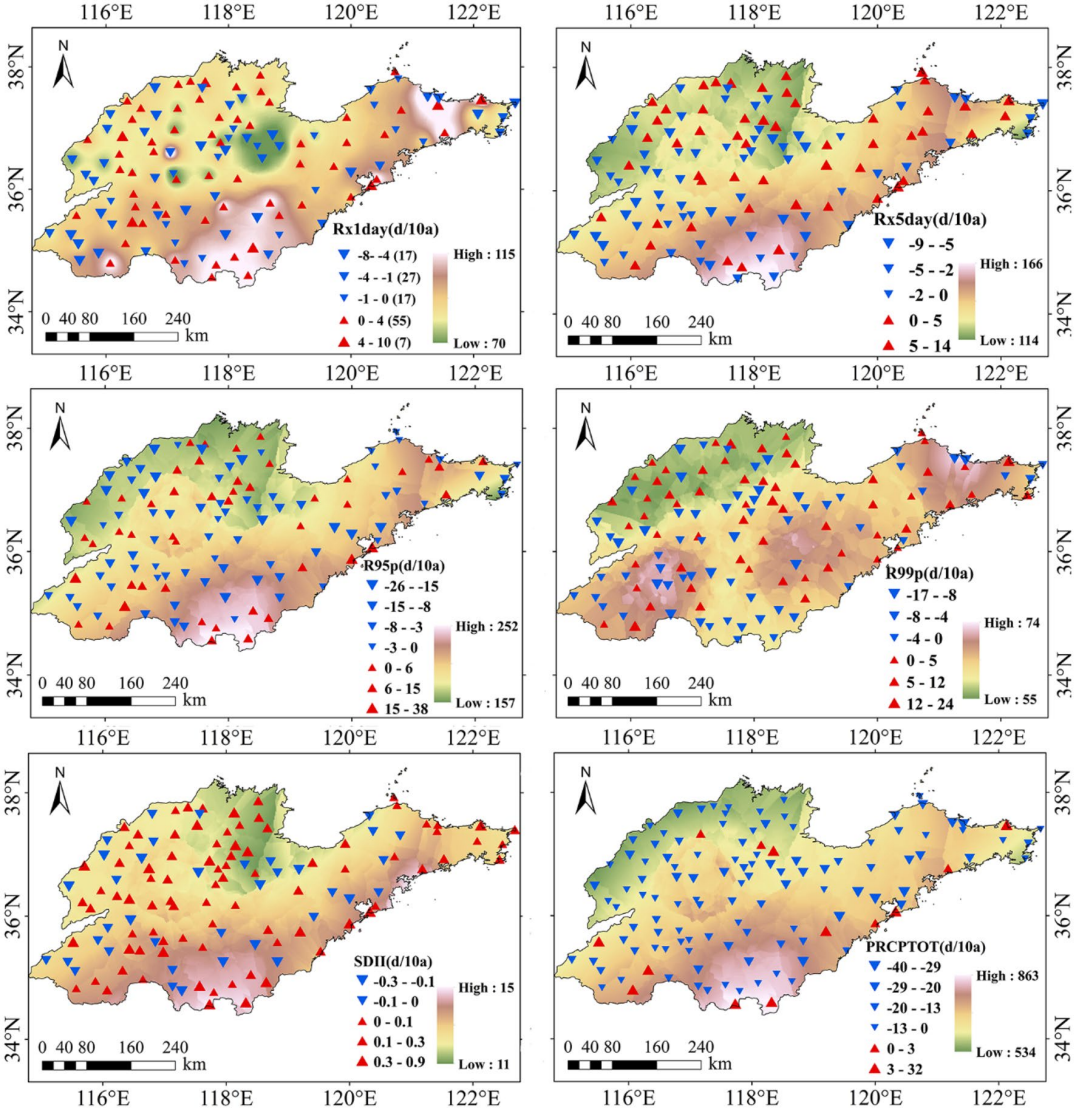
Temporal distribution of indices related to the number of precipitation days in Shandong from 1961 to 2015.

From 1961 to 2015, the extreme indices related to the number of precipitation days in the Shandong Province showed certain differences in spatial distribution. Kriging interpolation was applied to the core indices of 123 stations in the Shandong Province, and the continuous spatial distribution in the region was obtained, as shown in Fig. 4.

From the northwest to the south of Shandong Province, the value of CDD decreases from 73 to 45 days. From the south to the northeast of Shandong Province, the value of Rx1day decreases from 45 to 37 days. The proportion of stations with upward and downward trends in CDD were approximately 69% and 31%, respectively, and the variation trends of all of the sites passed the significance test. The spatial distributions of CWD, R10, R20, and Rnn were similar. From the northwest to the south of Shandong Province, the value of CWD increases from 3 to 5 days, the value of R10 increases from 14 to 24 days, the value of R20 increases from 7 to 13 days, the value of Rnn increases from 3 to 6 days, and the variation trends of all of the sites passed the significance test. The proportions of the stations with an upward and downward trend of CWD were about 17% and 83%, respectively. The proportions of stations with upward and downward trends of R10 were about 20% and 80%, respectively. The proportions of stations with upward and downward trends of R20 were about 24% and 76%, respectively. The proportions of sites with upward and downward trends of Rnn were about 33% and 67%, respectively.

### Mutation analysis of extreme precipitation

#### Indices related to the amount of precipitation

**Figure 5:**
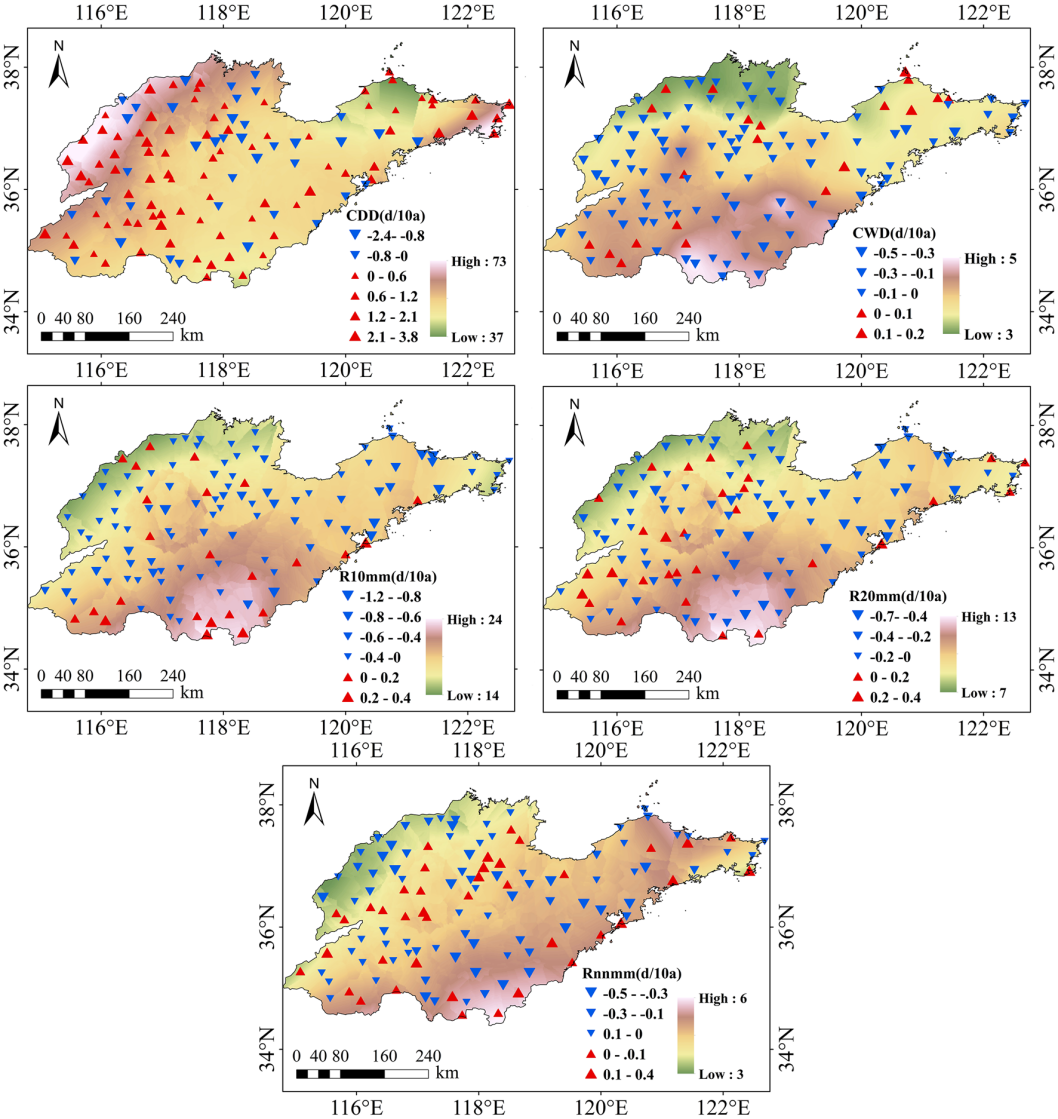
Mann–Kendall test and cumulative anomaly of indices related to the amount of precipitation in Shandong from 1961 to 2015.

The mutation characteristics of the indices related to the amount of precipitation in the Shandong Province are shown in Fig. 5. As shown in Fig. 5a, the results of Rx1day's M–K test showed that there were several intersections of the UF and UB curves in the confidence interval, including three from 1961 to 1965, three from 1992 to 1996, and one in 2014, respectively. The cumulative anomaly curve of Rx1day showed that the mutation years were 1965, 1975, 1989. From the Pettitt test, the mutation year of Rx1day was 1975. Considering the results of the above three statistical methods, Rx1day may have undergone from more to less mutations around 1975. As shown in Fig. 5b, the results of Rx5day's M–K test showed that the UF and UB curves had multiple intersections in the confidence interval, including two before and after 1964, three from 2002 to 2005, and two before and after 2014. The cumulative anomaly curve of Rx5day showed that the mutation years were 1965, 1978, 1992. The Pettitt test showed that the mutation year of Rx5day was 1978. Comprehensively, it is known that Rx5day may have undergone from more to less mutations around 1978. As shown in Fig. 5c, the M − K test results of R95p showed that the UF and UB curves had multiple intersections within the confidence interval, one after 1961, one in 1964, one from 2002 to 2003, and one from 2013 to 2014. The cumulative anomaly variation curve of R95p showed that the mutation years were 1965, 1975, 1989. The Pettitt test showed that the mutation year of R95p was 1978. Considering the results of the above three statistical methods, R95p may have undergone from more to less mutations around 1975. As shown in Fig. 5d, the M − K test results of R99p showed that the UF and UB curves had multiple intersections within the confidence interval, including two around 1964, one around 1993, and two around 2014. The cumulative anomaly curve of R99p was similar to that of R95p. The Pettitt test showed that the mutation year of R99p was 1975. In general, R99p may have undergone from more to less mutations around 1975. As shown in Fig. 5e, the M–K test results of SDII showed that there were several intersections of the UF and UB curves in the confidence interval, including three from 1994 to 1997 and two from 2014 to 2014. The variation in the cumulative anomaly curve of SDII showed that the mutation years were 1965, 1975, 1991. The Pettitt test showed that the mutation year of SDII was 1993. Thus, the mutation of SDII may have occurred from less to more from 1992 to 1993. As shown in Fig. 5f, the M–K test results of PRCPTOT showed that the UF and UB curves had multiple intersections in the confidence interval, including one in 1963, one from 1994 to 1995, and one in 2012. The variation of cumulative anomaly curve of PRCPTOT showed that the mutation years were 1964, 1975, 2002. The Pettitt test showed that the mutation year of PRCPTOT was 1975. Considering the results of the above three statistical methods, PRCPTOT may have undergone from less to more mutations around 1975.

#### Indices related to the amount of precipitation

**Figure 6:**
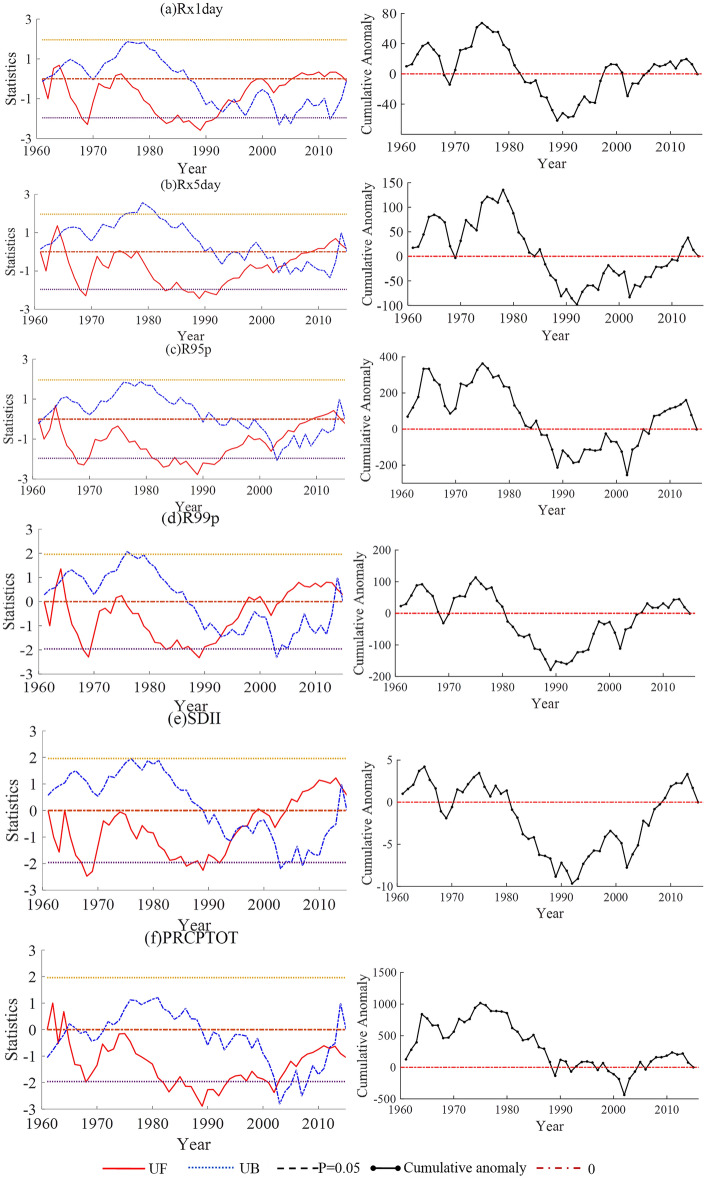
Temporal variations of indices related to the amount of precipitation in Shandong from 1961 to 2015.

The time variation trends of the extreme indices related to the amount of precipitation in the Shandong Province from 1961 to 2015 are shown in Table 2 and Fig. 6. The variation trends of all of the extreme precipitation indices did not pass the significance test at the 95% confidence level. Rx1day, Rx5day, R95p, and PRCPTOT showed a decreasing trend, and their climate tendency rates were − 0.16 mm/10a, − 0.28 mm/10a, − 2.60 mm/10a, and − 12.88 mm/10a, respectively. From the perspective of the 5-point moving average, Rx1day showed an oscillatory decline from the late 1960s to the 1980s, an upward trend of volatility after the 1980s, and a relatively stable downward trend after 2004. Before entering the twenty-first century, the changes of Rx5day tended to be consistent with those of Rx1day. After entering the twenty-first century, Rx5day tended to be almost stable, and there was almost no clear variation trend. The variation trends of R95p and PRCPTOT were basically the same, showing an oscillatory decline from the 1960s to the beginning of the twenty-first century, rising slightly after 2003, and then showing a weak downward trend. R99p and SDII showed upward trends, and their climate tendency rates were 0.69 mm/10a and 0.07 (mm/d)/10a, respectively. From the perspective of the 5-point moving average, R99p and SDII showed oscillatory declines from the 1960s to the 1980s, a fluctuating upward trend after the 1980s.

#### Indices related to the number of precipitation days

**Figure 7:**
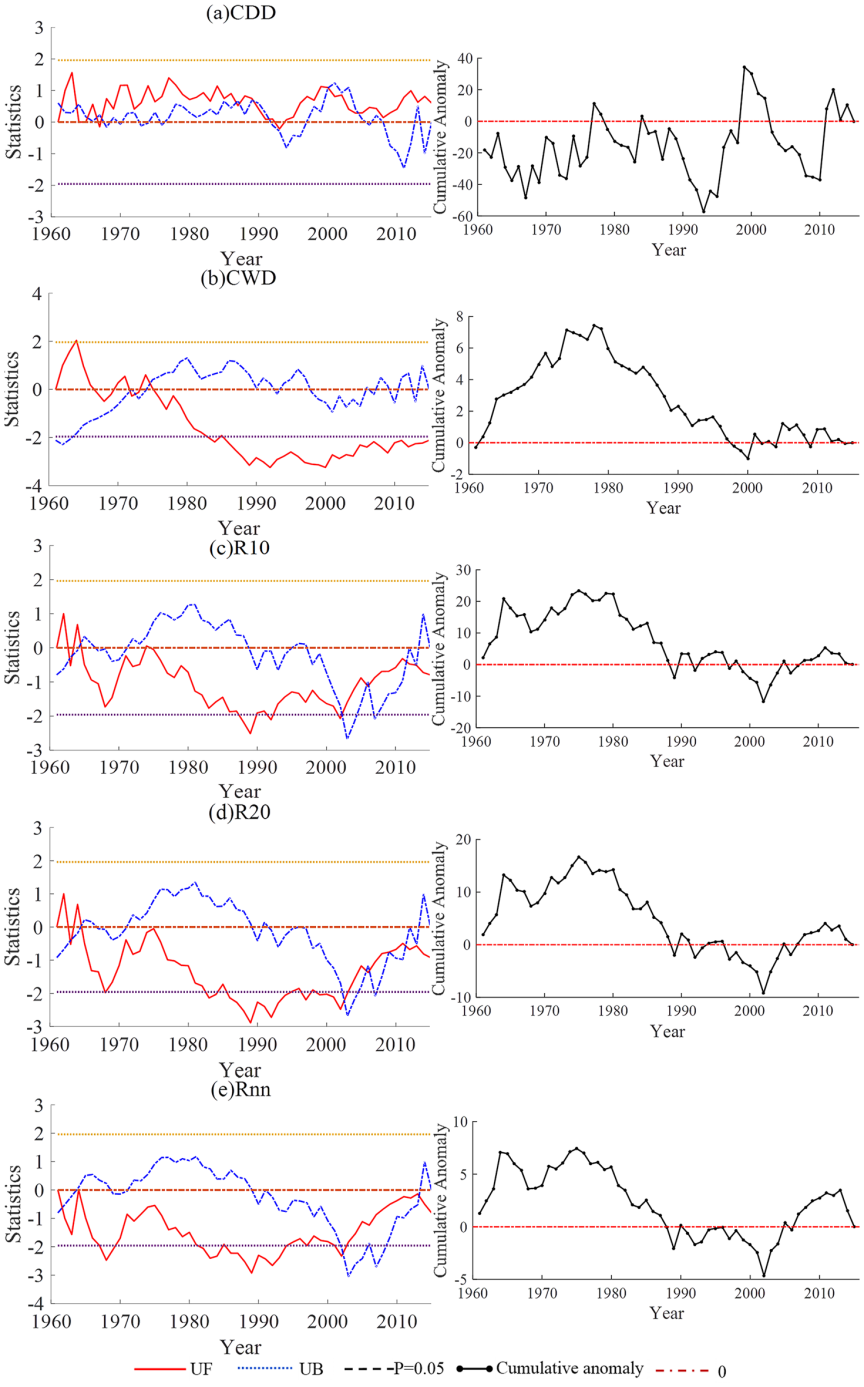
Mann–Kendall test and cumulative anomaly of indices of the number of precipitation days in Shandong from 1961 to 2015.

The mutation characteristics of the indices related to the number of precipitation days in the Shandong Province are shown in Fig. 7. As shown in Fig. 7a, the M − K test results of CDD showed that the UF and UB curves had multiple intersections in the confidence interval, including five from 1961 to 1968, two around 1987, one from 1989 to 1990, one from 1991 to 1992, one in 2000, and one from 2004 to 2005. The variation in the cumulative anomaly curve of CDD showed that the mutation years were 1977, 1993, 1999. The Pettitt test showed that the mutation year of CDD was 1993. Considering the results of the above three statistical methods, the CDD may have mutated from less to more around 1993. As shown in Fig. 7b, the M − K test results of CWD showed that the UF and UB curves had multiple intersections in the confidence interval, two around 1972, and one from 1974 to 1975. The cumulative anomaly curve of CWD showed that the mutation years were 1978, 2000. The Pettitt test showed that the mutation year of CWD was 1979. Considering the results of the above three statistical methods, the mutation in CWD may have occurred from more to less around 1978 to 1979. As shown in Fig. 7c, the M − K test results of R10 showed that there were several intersections of the UF and UB curves in the confidence interval, including two before and after 1963, one from 1964 to 1965, one from 2002 to 2003, two from 2006 to 2011, and two from 2011 to 2014. The cumulative anomaly curve of R10 showed that the mutation years were 1964, 1976, 1979, 2002. The Pettitt test showed that the mutation year of R10 was 1979. Considering the results of the above three statistical methods, the mutation in R10 may have occurred from more to less around 1978–1979. As shown in Fig. 7d, the M − K test results of R20 showed that the UF and UB curves had multiple intersection points within the confidence interval, two around 1963, one between 1964 and 1965, two around 2007, one in 2009, and two between 2011 and 2014. The cumulative anomaly curve of R20 showed that the mutation years were 1964, 1975, 2002. The Pettitt test found that the mutation year of R20 was 1975. In general, R20 may have undergone a mutation around 1975. As shown in Fig. 7e, the M − K test results for Rnn showed that the UF and UB curves had multiple intersections within the confidence interval, one in 1961 − 1962, and one from 2013 to 2014. The cumulative anomaly curve of Rnn showed that the mutation years were 1964, 1975, 2002. The Pettitt test found that the Rnn mutation year was 1975. In general, Rnn may have a mutation from more to less around 1975.

In summary, the mutation time and tendency of the extreme precipitation indices in the Shandong Province are shown in Table 3^31^. The results of the two studies are basically the same. Among the 11 core indices of extreme precipitation in the Shandong Province, the mutation of nine core indices may have occurred from more to less in the late 1970s, and only two occurred from less to more mutation around 1993. According to the variation trends of the elements before and after the mutation of the core index, the precipitation in the Shandong Province has gradually decreased, and the drought conditions have gradually increased.

**Table 3:** Mutation time and trends of extreme precipitation indices in Shandong Province.

Indices	Precipitation indices						Indices of the number of precipitation days				
	Rx1day	Rx5day	R95p	R99p	SDII	PRCPTOT	CDD	CWD	R10	R20	Rnn
Mutation time1^a^	1975	1978	1975	1975	1992 − 1993	1975	1993	1978 − 1979	1978 − 1979	1975	1975
Mutation time2^b^	1965	1978	1965	1965	1993	1975	1993	1978	1975	1975	1975
Tendency1^1^	**↓**	**↓**	**↓**	**↓**	**↑**	**↓**	**↑**	**↓**	**↓**	**↓**	**↓**
Tendency2^2^	**↓**	**↓**	**↓**	**↓**	**↑**	**↓**	**↑**	**↓**	**↓**	**↓**	**↓**

^a^Mutation time1 and Tendency1 represents the results of extreme precipitation indices in this study.

^b^Mutation time2 and Tendency2 represent the results of extreme precipitation indices in the coastal areas of Huanghuai, China (including Shandong Province).

### Periodic analysis of extreme precipitation

#### Indices related to the amount of precipitation

**Figure 8:**
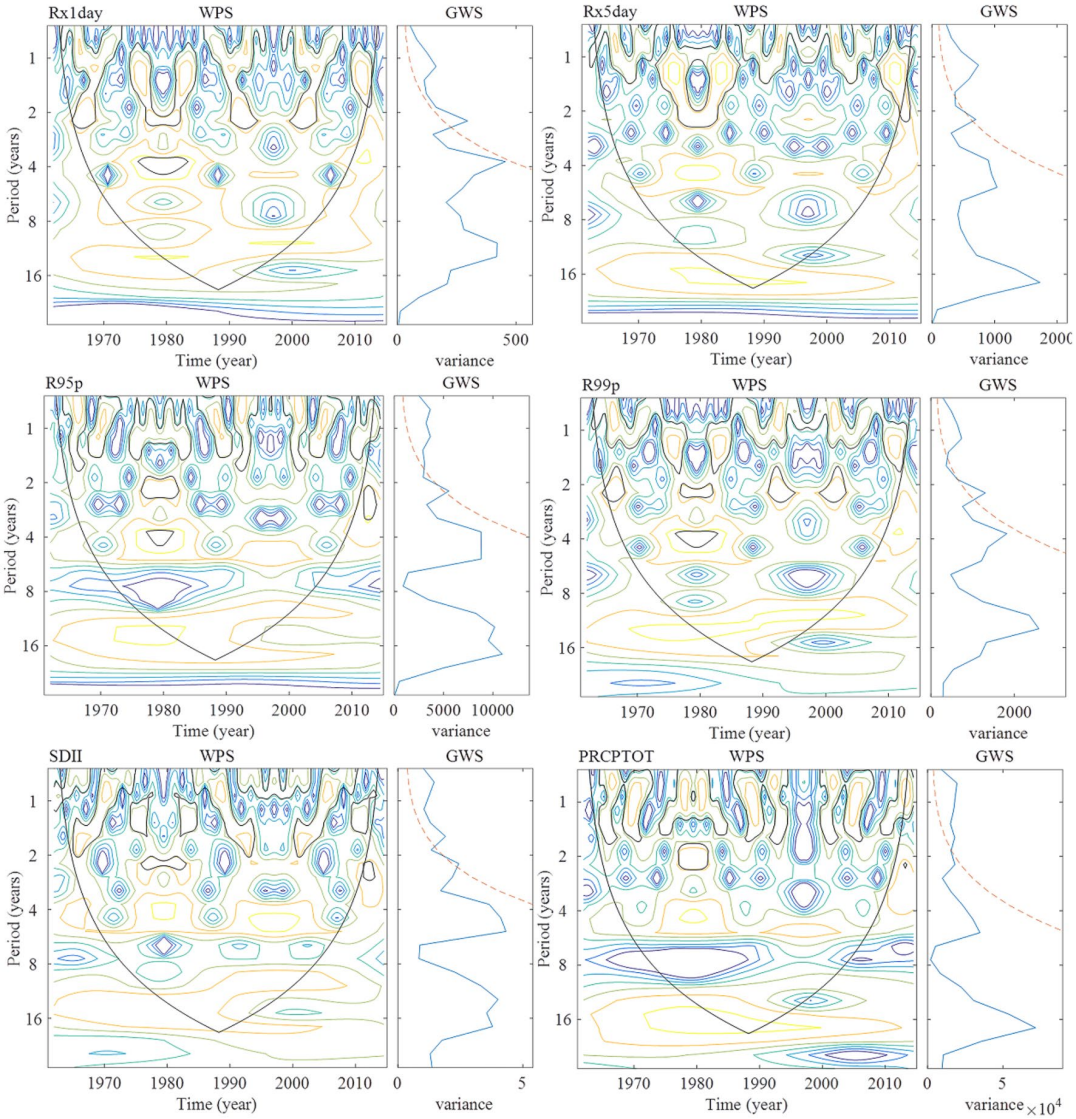
Wavelet power spectrum and global wavelet spectrum for the time series of indices related to the amount of precipitation in Shandong from 1961 to 2015 (The range surrounded by the black outline in WPS refers to passing 95% of the significance level, the red dashed line in GWS is the 95% confidence spectrum ).

The wavelet power spectrum (WPS) and global wavelet power (GWP) of the time series of the indices related to the amount of precipitation in the Shandong Province are shown in Fig. 8. Rx1day had a main period of around 2.2 years, a significant period of 1.1 years, and non-significant periods of 3.7 and 10.6 years. Rx5day had a main period of around 1.1 years, a significant period of around 2.2 years, and a non-significant period of around 5.3 years. R95p had a main period of around 2.2 years, a significant period of around 0.8 and 1.1 years, and non-significant periods of around 5.3 and 12.6 years. R99p has a main period of around 2.2 years, a significant period of around 1.1 years, and non-significant periods of around 3.7 and 12.6 years. SDII had a main period around 2.2 years, significant periods of around 0.8 and 1.6 years, and a non-significant period of 5.3 years. PRCPTOT had a main period of around 0.8 years, a significant period of 1.6 years, and non-significant periods of around 2.2 and 5.3 years.

#### Indices related to the number of precipitation days

**Figure 9:**
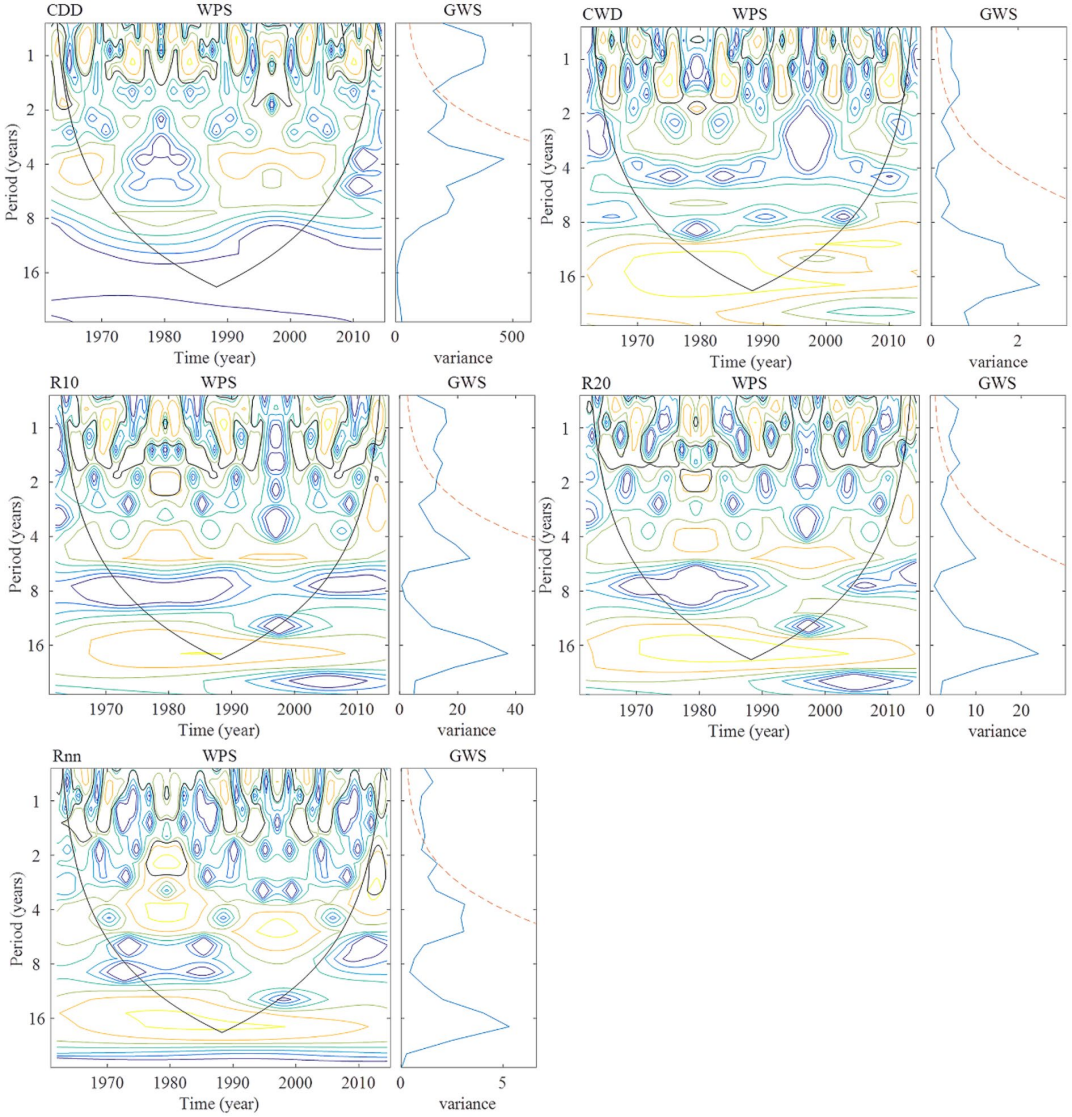
Wavelet power and global wavelet spectra for the time series of the indices related to the number of precipitation days in Shandong from 1961 to 2015 (The range surrounded by the black outline in WPS refers to passing 95% of the significance level, the red dashed line in GWS is the 95% confidence spectrum ).

The wavelet power spectrum (WPS) and global wavelet power (GWP) of the time series of the indices related to the number of precipitation day in the Shandong Province are shown in Fig. 9. CDD has a main period of around 0.9 years, and non-significant periods of around 1.9, 3.7, and 6.3 years. CWD had a main period of around 1.6 years, a significant period of around 0.8 years, and a non-significant period of around 3.1 and 6.3 years. R10 had a main period of around 0.9 years, a significant period of around 1.6 years, and a non-significant period around 5.3 years. R20 has a main period around 1.6 years, a significant period of around 0.8 years, and a non-significant period of around 5.3 years. Rnn had a main period of around 2.2 years, significant periods of around 0.8 and 1.6 years, and non-significant periods of around 3.7 and 5.3 years.

In summary, the main period of each extreme precipitation index in the Shandong Province is shown in Table 4^31^. The Indices related to the amount of precipitation of the two research results are basically consistent, while the indices related to the number of precipitation days in this study were relatively small. The results show that precipitation events (CWD), drought events (CDD) and extreme precipitation events (the Period of R10, R20, and Rnn is shortened) occur more frequently in Shandong Province under the condition of basically the same precipitation intensity (R95p, R99p, SDII).

**Table 4:** Primary period of extreme precipitation indices in Shandong Province.

Indices	Indices related to the amount of precipitation						Indices related to the number of precipitation days				
	Rx1day	Rx5day	R95p	R99p	SDII	PRCPTOT	CDD	CWD	R10	R20	Rnn
Period1^a^	2.2	1.1	2.2	2.2	2.2	2.2	0.9	1.6	0.9	1.6	2.2
Period2^b^	2.3	2.3	2.3	2.3	2.3	4.7	2.8	3.9	2.8	4.7	4.7
$\frac{\mathrm{Period}1}{\mathrm{Period}2}$	1.0	0.5	1.0	1.0	1.0	0.5	0.3	0.4	0.3	0.3	0.5

^a^Period1 represents the primary period of extreme precipitation indices in this study.

^b^Period2 represents the primary period of extreme precipitation indices in the coastal areas of Huanghuai, China (including Shandong Province).

## Conclusions

The variation trends of extreme events in China have evident regional characteristics. In this study, based on the daily precipitation data of 123 meteorological stations in Shandong Province from 1961 to 2015, 11 extreme precipitation indices were selected to analyze the spatiotemporal variations and statistical characteristics of extreme precipitation in the Shandong Province. The main conclusions are given as follows:

1. From the perspective of a time series, Rx1day, Rx5day, R95p, PRCPTOT, CWD, R10, R20, and Rnn showed a downward trend in Shandong in the past 55 years, among which the PRCPTOT decreased most significantly and Rnn decreased the least. However, the extreme precipitation index showed upward trends with varying degrees after the 1980s, which was consistent with the extreme precipitation event results worldwide and in China^32,33^. This shows that extreme precipitation events have undergone an increasing trend, while the increase in CDD and the decrease in CWD also indicated that the drought events underwent an increasing trend in Shandong Province.
2. From the perspective of spatial change, the indices basically showed a trend of increasing gradually from north to south. The main periods of extreme precipitation time series in the Shandong area were basically similar ranging from 0.9 to 2.2 years, and there were no significant inter-decadal periodic oscillations. The mutation time of extreme precipitation indices mainly occurred in the late 1970s and early 1990s.The results of the study on the abrupt characteristics of precipitation and the extreme events in China showed that the precipitation in China underwent significant changes in the 1970s and 1990s, respectively.

For the study of the temporal and spatial variations of extreme precipitation in the Shandong Province, there are still many areas worth exploring in the future. When dealing with extreme precipitation to climate change, some engineering measures and non-engineering measures should be taken to do a good job in regional water resources management and agricultural development planning. The specific implementation measures need further research. In the future research, we must understand the extreme precipitation disasters in Shandong and build a model to predict future trends. This will allow us to accurately judge the hazards of disaster-causing factors, evaluate the vulnerability of the ecological environment and human society, identify high-risk areas of extreme precipitation, implement accurate risk management countermeasures, and build a comprehensive risk prevention system of the social − ecological system in Shandong.

## Data Availability

Data and material used were derived from public domain resources.
