# Author Correction: Dominant change pattern of extreme precipitation and its potential causes in Shandong Province, China

**DOI:** 10.1038/s41598-022-26830-7

**Published:** 2022-12-23

**Authors:** Jun Xia, Xu‑yang Yang, Jian Liu, Mingsen Wang, Jiake Li

**Affiliations:** 1 School of Water Conservancy and Hydropower, Xi’an University of Technology, Xi’an, China; 2 State Key Laboratory of Water Resources and Hydropower Engineering Science, School of Water Resources and Hydropower Engineering, Wuhan University, Wuhan, China; 3 Post‑Doctoral Research Center, Shandong Provincial Academician Workstation, Water Resources Research Institute of Shandong Province, Jinan, China

Correction to: *Scientific Reports* 10.1038/s41598-022-04905-9, published online 17 January 2022

In the original version of this Article, Affiliation 1 and 2 were not listed in the correct order. The correct affiliations are listed below:

Affiliation 1:

School of Water Conservancy and Hydropower, Xi’an University of Technology, Xi’an, China.

Affiliation 2:

State Key Laboratory of Water Resources and Hydropower Engineering Science, School of Water Resources and Hydropower Engineering, Wuhan University, Wuhan, China.

The original Article has been corrected.

